# Hydrodynamic function and spring constant calibration of FluidFM micropipette cantilevers

**DOI:** 10.1038/s41378-023-00629-6

**Published:** 2024-02-18

**Authors:** Attila Bonyár, Ágoston G. Nagy, Hans Gunstheimer, Gotthold Fläschner, Robert Horvath

**Affiliations:** 1 Department of Electronics Technology, Faculty of Electrical Engineering and Informatics, Budapest University of Technology and Economics, Budapest, Hungary; 2 Nanobiosensorics Laboratory, Institute of Technical Physics and Materials Science, Centre for Energy Research, HUN-REN, Budapest, Hungary; 3 Nanosurf AG, Gräubernstrasse 12, CH-4410 Liestal, Switzerland

**Keywords:** Electrical and electronic engineering, NEMS, Nanosensors, Nanofluidics

## Abstract

Fluidic force microscopy (FluidFM) fuses the force sensitivity of atomic force microscopy with the manipulation capabilities of microfluidics by using microfabricated cantilevers with embedded fluidic channels. This innovation initiated new research and development directions in biology, biophysics, and material science. To acquire reliable and reproducible data, the calibration of the force sensor is crucial. Importantly, the hollow FluidFM cantilevers contain a row of parallel pillars inside a rectangular beam. The precise spring constant calibration of the internally structured cantilever is far from trivial, and existing methods generally assume simplifications that are not applicable to these special types of cantilevers. In addition, the Sader method, which is currently implemented by the FluidFM community, relies on the precise measurement of the quality factor, which renders the calibration of the spring constant sensitive to noise. In this study, the hydrodynamic function of these special types of hollow cantilevers was experimentally determined with different instruments. Based on the hydrodynamic function, a novel spring constant calibration method was adapted, which relied only on the two resonance frequencies of the cantilever, measured in air and in a liquid. Based on these results, our proposed method can be successfully used for the reliable, noise-free calibration of hollow FluidFM cantilevers.

## Introduction

**Fig. 1:**
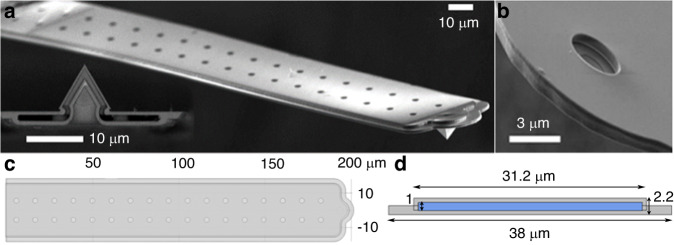
Structure of the FluidFM micropipette cantilevers. **a** Scanning electron microscopy (SEM) image of a FluidFM cantilever with a nanopipette head. **b** SEM image of a micropipette head with a circular aperture. **c** 3D reconstruction of the micropipette cantilever in the COMSOL Multiphysics environment. **d** Cross-section of the same 3D model, with the channel highlighted in blue

Fluidic force microscopy (FluidFM) is an extension of atomic force microscopy (AFM) with a nanofluidic system consisting of a pressure-controlled refillable fluid reservoir connected to special microfabricated cantilevers with integrated fluidic channels^1^. Different types of FluidFM cantilevers are available for a large variety of applications, including 2D and 3D printing^2–4^, the colloidal probe technique^5–7^, injection/extraction of liquids into/from living cells^1,8^, and single-cell force spectroscopy (SCFS)^9–14^. The tipless micropipette cantilevers (Fig. 1) are primarily designed for the latter purpose; the hollow beam contains a flat, circular aperture (with 2, 4, or 8 μm diameter) optimized for the easy attachment of colloidal particles or living cells^7,13^.

In the above applications, the manipulation of living cells, beads, and injected/extracted liquids is conducted under the precise force control of FluidFM cantilevers by measuring their deflection with the help of a laser beam reflected back from the cantilever^15^. Since the fluidic channel inside the cantilever is connected to a pressure controller system, the user has the capability to precisely control the injection/extraction of the liquids with the use of nanopipettes (hollow cantilevers with a pyramidal tip) or with the attachment/detachment of colloidal beads or living cells to micropipettes by simply changing the pressure inside the hollow cantilevers.

Single-cell biology has particularly benefited from the application of FluidFM. The manipulation of mitochondria^16^, fungi cells^17^, and single-cell cytoplasmic extraction for mass spectrometry measurements^8^ have all been demonstrated. Single-cell temporal transcriptomics from tiny FluidFM biopsies (LiveSeq) could address a broad range of basic biological questions. The method introduced recently by the groups of Vorholt and Deplancke transformed scRNA-seq from an endpoint to a temporal analysis workflow with the help of FluidFM^18,19^.

A particularly interesting and yet unexplored application of FluidFM is to precisely measure the mass of objects (nanoparticles, beads, living cells) attached to the cantilever^20–22^, potentially recording the nanoscale motion^23–25^ of these objects upon interaction with various chemical or biological agents.

Notably, the above applications can be significantly scaled up by combining FluidFM technology with a large-area motorized sample stage and computer automatization. These robotic FluidFM setups were successfully used for mm–cm scale printing of live cell patterns^4^ and to first measure the adhesion force distributions of large cell populations^13,26^ and cancer cells adhering to compact epithelial layers^27^. Robotic FluidFM was also used to calibrate the signal of other high-throughput devices with respect to adhesion force and energy; these devices included large-area single-cell biosensors^13^ and computer-controlled micropipettes^7^. Clearly, the precise force calibration of FluidFM itself has crucial importance in these directions when FluidFM and an indirect force measuring technology are combined.

In our recent work, the effect of the special hollow cantilever geometry on the calibration of the spring constant (*k*) and optical lever sensitivity of micropipette cantilevers was investigated^14^. It was found that by using the FluidFM instrument, which utilized classical thermal noise tuning and the Sader method for spring constant calibration, the obtained spring constants had an error of 20%^14^. This error is unacceptably high, considering that *k* directly scales and influences both the measured force and elastic modulus during force spectroscopy measurements or mass measurements^14^. The experienced error in the calibration of the spring constant was traced back to the improper determination of the *Q*-factor due to high noise levels. Compared with normal AFM, other groups also found that the Sader method had uncertainties of ~20% when performing the calibration in liquid^28^.

In this current study, a different calibration strategy is provided that eliminates the need to determine the *Q*-factor of the resonance frequencies and relies only on the determination of the first modes of the resonance frequencies measured in air and water.

## Theory

The current FluidFM setups implemented the Sader method^29–34^, which relies on the geometrical properties of the cantilever, such as its width (*b*), length (*L*) and effective mass (${M}_{{\rm {e}}}$), along with its fundamental resonance frequency (${\omega }_{{\rm {f}}}$) and quality factor (*Q*), measured in a fluid with a density of ${\rho }_{\rm {{f}}}$, as in Eq. (1).

1 $$k={M}_{{\rm {e}}}\frac{\pi }{4}{\rho }_{{\rm {f}}}{b}^{2}{LQ}{\varGamma }_{\rm {{i}}}({\omega }_{{\rm {f}}}){{\omega }_{{\rm {f}}}}^{2}$$

It also uses the imaginary part of the hydrodynamic function ($\varGamma (\omega ))$. The real (${\varGamma }_{{\rm {r}}}$) and imaginary (${\varGamma }_{{\rm {i}}}$) parts of the hydrodynamic function represent the effect of the surrounding pressure on the cantilever^35^, considering the added mass and damping stiffness per unit length of the cantilever^36–38^. Instead of its full numerical form^29^ $\varGamma (\omega )$ is often approximated with two semianalytical functions, Eqs. (2) and (3). Here, ${a}_{1}$,${a}_{2}$ and ${b}_{1}$,$\,{b}_{2}$ are real and imaginary regression coefficients^35,37,39^, while $\delta$ is the thickness of the thin viscous layer surrounding the cantilever in which the velocity has been reduced by a factor of 1/*e*^37^.

2 $${\varGamma }_{r}\left(\omega \right)={a}_{1}+{a}_{2}\left(\frac{\delta }{b}\right){=a}_{1}+\frac{{a}_{2}}{\sqrt{\mathrm{Re}}}$$

3 $${\varGamma }_{i}\left(\omega \right)={b}_{1}\left(\frac{\delta }{b}\right)+{b}_{2}{\left(\frac{\delta }{b}\right)}^{2}=\frac{{b}_{1}}{\sqrt{\mathrm{Re}}}+\frac{{b}_{2}}{\mathrm{Re}}$$

Here, we use the original formulation of the Reynolds number Re^29,32^ and ${\varGamma }_{{\rm {r}}}\left({\omega }_{{{\rm {f}}}n}\right)$^40,41^, as defined in Eqs. (4) and (5), where $\mu$ is the dynamic viscosity of the medium and ${\omega }_{{{\rm {a}}}n}$ and ${\omega }_{{{\rm {f}}}n}$ refer to the *n*th normal mode of the resonance frequencies measured in air (a) and in a fluid (f), respectively.

4 $$\mathrm{Re}({\omega }_{{{\rm {f}}}n})=\frac{{\rho }_{{\rm {f}}}{b}^{2}{\omega }_{{{\rm {f}}}n}}{4\mu }$$

5 $${\varGamma }_{{\rm {r}}}\left({{\omega }_{{{\rm {a}}n}},\omega }_{{{\rm {f}}n}}\right)=\frac{4{\rho }_{{\rm {c}}}h\left(\frac{{\omega }_{{{\rm {a}}n}}^{2}}{{\omega }_{{{\rm {f}}n}}^{2}}-1\right)}{{\rho }_{{\rm {f}}}\pi b}$$

There are three major issues with the direct implementation of the Sader method for the calibration of FluidFM micropipette cantilevers. First, it depends heavily on the precise determination of the *Q*-factor, which could be difficult to measure, especially in viscous environments^42^. As extensively demonstrated in our previous work, the high variation in the obtained *k* of micropipette cantilevers originated directly from the position dependency of the detection laser along the length of the cantilever and the improper determination of the *Q*-factor due to high noise levels at some points^14^. Second, the original hydrodynamic function given by Sader^29^ is theoretically applicable for rectangular beams of infinite length, whose width (*b*) is greater than its thickness (*h*)^6,37^. Although Sader later extended the method to other arbitrary shapes^32^ and several commercially available, nonrectangular AFM cantilever types^33^, the special, hollow structure of the FluidFM micropipette cantilever has not yet been investigated. Third, the normalized effective mass (${M}_{{\rm {e}}}$) in Eq. (1) is also a geometry-dependent factor. Sader found that this value was 0.243 for long rectangular cantilevers^43^; thus, ${M}_{{\rm {e}}}$ is often lumped together with π/4 and given as 0.1906 in simplified versions of Eq. (1)^30,32,33^. However, ${M}_{{\rm {e}}}$ has not yet been directly determined for FluidFM micropipette cantilevers.

Notably, the robotic FluidFM setup (FluidFM OMNIUM) implemented the Sader method and presumed that the original hydrodynamic function and normalized effective mass for infinite rectangular beams would be applicable for micropipette cantilevers; however, these were consequential simplifications.

In a recent paper, Payam et al. published an alternative formula for the calibration of spring constants (Eq. (6)), which successfully eliminated the dependence on the *Q*-factor^42^. This method only required the measurement of two resonance frequencies, one in air (${\omega }_{{\rm {a}}}$) and another in a fluid (${\omega }_{{\rm {f}}}$) (preferably water), in addition to some geometrical (width (*b*) and length (*L*) of the cantilever) and material (density (${\rho }_{{\rm {f}}}$) and viscosity ($\eta$) of the fluid) properties.

6 $$k=\frac{{\omega }_{{\rm {f}}1}^{2}\pi {{\rm {a}}}_{1}{\rho }_{{\rm {f}}}b+2{\omega }_{{\rm {f}}1}^{3/2}\pi {a}_{2}\sqrt{{\rho }_{\rm {{f}}}\eta }}{16({\omega }_{{\rm {a}}1}^{2}-{\omega }_{{\rm {f}}1}^{2})}{bL}{\omega }_{{\rm {a}}1}^{2}$$

Since the FluidFM cantilever is intended to be used in fluidic environments, this calibration method is rather convenient and straightforward for this instrument. However, Eq. (6) still relies on the hydrodynamic function in the form of the two real regression coefficients, ${{a}}_{1}$ and ${a}_{2}$ from Eq. (2), which is not available for FluidFM micropipette cantilevers, as mentioned before. Although the regression coefficients introduced by Maali et al. (*a*_1_ = 1.0553; *a*_2_ = 3.7997; *b*_1_ = 3.8018; *b*_2_ = 2.7364)^37^ are widely used in cases where an infinitely long beam simplification is acceptable^39^, the applicability of these constants for hollow FluidFM cantilevers has not yet been experimentally validated.

To obtain the missing constants and, therefore, estimate the hydrodynamic function of this special cantilever type, another formula is used, deduced from the Euler‒Bernoulli partial differential equation, and provided by Payam et al. in their recent study^42^. Equation (7) can be obtained by substituting Eqs. (4) and (5) into Eq. (2) and connect the regression coefficients with the general angular resonance frequencies of the cantilever in air (${\omega }_{{{\rm {an}}}}$) and in a fluid (${\omega }_{{{\rm {fn}}}}$) for any given mode (*n*). In addition to the previously listed geometrical and material properties, the areal mass density of the cantilever (${\rho }_{\rm {{c}}}h$) is also needed.

7 $${\omega }_{{{\rm {fn}}}}^{2}\left(\frac{\pi {a}_{1}{\rho }_{{\rm {f}}}b}{4{\rho }_{{\rm {c}}}h}+1\right)+{\omega }_{{{\rm {fn}}}}^{\frac{3}{2}}\left(\frac{\pi {a}_{2}\sqrt{\eta {\rho }_{{\rm {f}}}}}{2{\rho }_{{\rm {c}}}h}\right)={\omega }_{{{\rm {an}}}}^{2}$$

Only the resonance frequencies of the first two modes in air and water are needed to solve the system of equations for ${a}_{1}$ and ${a}_{2}$. The resulting equations are given as Eqs. (8) and (9).

8 $${a}_{1}=\frac{4{\rho }_{{\rm {c}}}h\left({\omega }_{{\rm {f}}1}^{\frac{3}{2}}\left({\omega }_{{\rm {f}}2}^{2}-{\omega }_{{\rm {a}}2}^{2}\right)+{\omega }_{f2}^{\frac{3}{2}}\left({\omega }_{{\rm {a}}1}^{2}-{\omega }_{{\rm {f}}1}^{2}\right)\right)}{b{\rho }_{{\rm {f}}}\pi \left({\omega }_{{\rm {f}}1}^{2}{\omega }_{{\rm {f}}2}^{\frac{3}{2}}-{\omega }_{{\rm {f}}1}^{\frac{3}{2}}{\omega }_{{\rm {f}}2}^{2}\right)}$$

9 $${a}_{2}=\frac{2{\rho }_{{\rm {c}}}h\left({\omega }_{{\rm {a}}2}^{2}{\omega }_{{\rm {f}}1}^{2}-{\omega }_{{\rm {a}}1}^{2}{\omega }_{{\rm {f}}2}^{2}\right)}{\pi \sqrt{\eta {\rho }_{{\rm {f}}}}\left({\omega }_{{\rm {f}}1}^{2}{\omega }_{{\rm {f}}2}^{\frac{3}{2}}-{\omega }_{{\rm {f}}1}^{\frac{3}{2}}{\omega }_{{\rm {f}}2}^{2}\right)}$$

With these regression coefficients and the geometrical parameters of the cantilever (*L*, *b*, $h$, ${\rho }_{{\rm {c}}}$), Eq. (6) can be used to obtain the spring constant.

Note that although the first two modes of the resonance frequencies are required to obtain the hydrodynamic function and its regression coefficients (${a}_{1}$ and ${a}_{2}$), if these are already known, then only the first modes measured in air and water are needed for the spring constant calibration (Eq. (6)).

To obtain the imaginary part of the hydrodynamic function, the two values of ${\varGamma }_{{\rm {i}}}\left(\mathrm{Re}\right)$, corresponding to Re values (Eq. (4)) in air and water can be calculated by using the resonance frequencies and their quality factors as in Eq. (10).

10 $${\varGamma }_{{\rm {i}}}\left({\omega }_{{{\rm {f}}n}}\right)=\frac{\frac{4{\rho }_{{\rm {c}}}h}{{\rho }_{{\rm {f}}}\pi b}+{\varGamma }_{\rm {{r}}}\left({\omega }_{{{\rm {f}}n}}\right)}{{Q}_{{{\rm {f}}n}}}$$

Subsequently, a system of equations can be constructed based on Eq. (2) for the ${\varGamma }_{{\rm {i}}}\left(\mathrm{Re}\right)$ pairs that can be solved for ${b}_{1}$ and ${b}_{2}$. These regression coefficients and the imaginary part of the hydrodynamic function are not needed for this calibration strategy, and we noted this possibility for the sake of completeness.

In the next sections, we will experimentally demonstrate how this approach can be used for the spring constant calibration of FluidFM cantilevers.

## Experimental

### FluidFM setup

A robotic FluidFM setup (FluidFM OMNIUM) from Cytosurge AG (CH) was used to record the first-mode resonance peaks from FluidFM micropipette cantilevers. The setup combined a FluidFM measuring head and pressure controller system with a motorized stage capable of manipulating the samples over mm-cm scale areas in a highly automatized manner^4,13,26,27^. To note, the instrument was called FluidFM BOT in earlier works^4,13^.

### Measurement of higher frequency modes

For the calculation of the regression and spring constants, the higher flexural mode resonances of a FluidFM micropipette (2 µm aperture, *k*_nom_ = 2 N/m) were measured using a DriveAFM from Nanosurf AG (CH). The instrument used photothermal excitation to directly excite the cantilever. Photothermal excitation allowed clean and stable actuation in air and liquids, avoiding the “forest of peaks” known from piezo-acoustic excitation^44^. With the AFM’s low noise controller (CX controller), the first three modes of the FluidFM micropipette in air and liquid were measured. An acoustic enclosure (AE550) with active temperature control (TEC controller) was used in combination with a damping table (Isostage300) to create an environment with low external disturbance at a temperature of 21.01 ± 0.05 °C. Nanosurf’s PicoBalance Software was used to acquire thermal tuning and frequency sweep data. The measurements were performed in air and ultrapure water filled in a polystyrene Petri dish (Falcon).

## Results and discussion

### Determination of the hydrodynamic function, the complete calibration method and its comparison with the Sader method

**Fig. 2:**
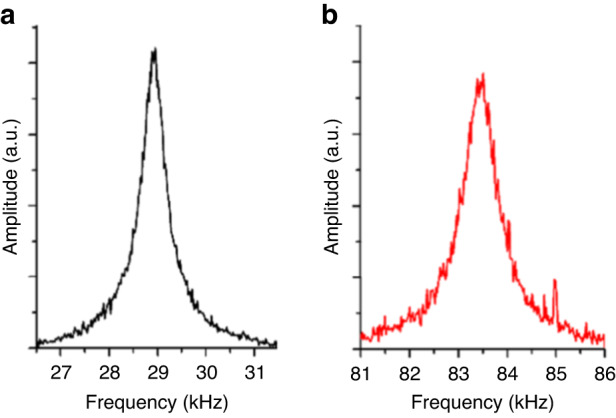
Raw thermal noise amplitude spectra of a FluidFM micropipette cantilever with an 8 µm aperture. **a** First mode, measured in water (channel filled with water). **b** First mode, measured in air (channel filled with water). The amplitude is given on an arbitrary scale (measured by the robotic FluidFM system)

Examples of the first-mode resonance peaks of FluidFM micropipette cantilevers measured in water (a) and air (b) with the robotic FluidFM instrument are shown in Fig. 2.

**Fig. 3:**
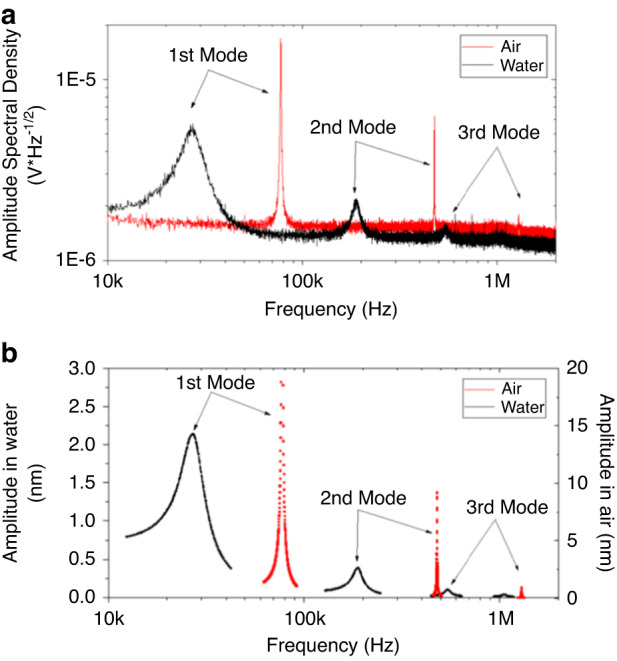
Tuning spectra of a FluidFM micropipette cantilever in air and water (channel filled with water), measured with a Nanosurf Drive AFM. The spectra were obtained with **a** thermal tuning and **b** the company’s CleanDrive Technology. In the latter case, the amplitude of the spectrum measured in water was multiplied by 5 for the sake of comparability. The spectra correspond with the data presented in Table 1

Based on 5 cantilevers, the first resonance peak varied between 70–84 kHz in air and 23–29 kHz in water. Notably, the high noise superposed on the peak, especially measured in air, which affected the determination of the *Q*-factor and, in turn, the obtained spring constant, as discussed in our previous paper^14^. Since the bandwidth of the detector in the robotic FluidFM instrument was limited by the sampling frequency of the AD converter and was ~170 kHz, the determination of the second modes of resonances was not possible with this system. For this reason, we experimentally determined the resonance frequencies and the hydrodynamic function of the micropipette cantilevers with a Nanosurf DriveAFM. Additionally, because of the stochastic nature of thermal noise, substantially longer averaging was needed to obtain a similarly accurate *Q*-factor fit value as could be obtained from directly actuating the cantilever and measuring its frequency-dependent response. Equipped with a high bandwidth detection system and CleanDrive Technology (photothermal excitation), ultralow noise spectra were acquired, as demonstrated in Fig. 3. The channel of the cantilever was filled with water for both measurements.

**Fig. 4:**
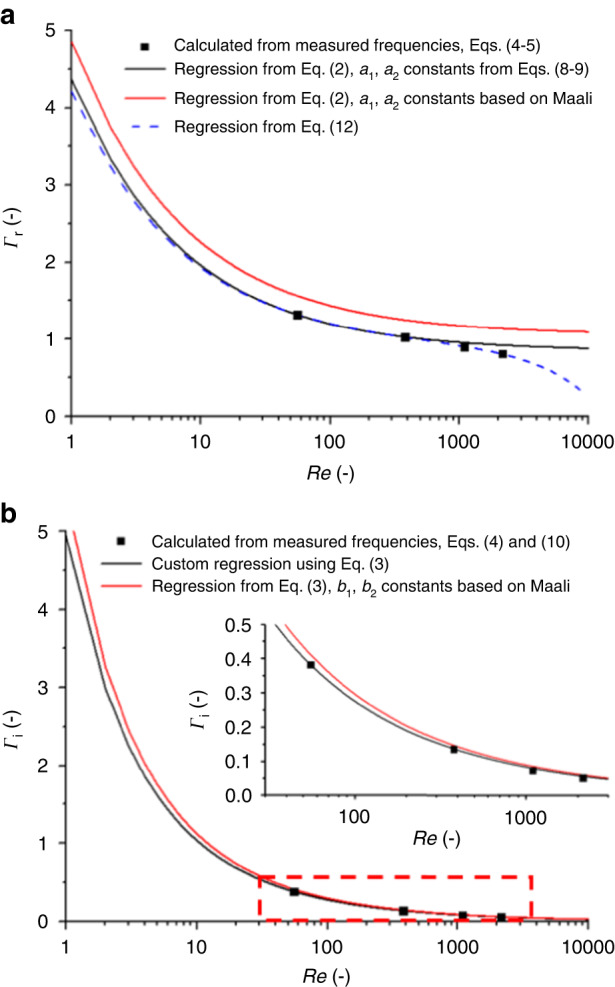
Comparison of hydrodinamic functions obtained by different calculation approaches. Real (**a**) and imaginary (**b**) parts of the hydrodynamic function for the measured cantilever (black) and the theoretical response for bulk, infinite slabs based on the Maali regression coefficients (red)

Based on the resonance peak positions of the first two modes in air and water, the hydrodynamic function and the spring constant of the cantilever could be determined by using Eqs. (6)–(9), as discussed in the section “Experimental”. The results are listed in Table 1, while the real and imaginary parts of the hydrodynamic function are shown in Fig. 4.

**Table 1:** The resonance peaks (in air and water), geometrical properties, obtained hydrodynamic function, and spring constant determined for a real cantilever based on measurements

	Parameters	Measured values
Resonance peaks	*f*_a1_ [kHz]	77.07
	*f*_a2_ [kHz]	475.91
	*f*_f1_ [kHz]	27.40
	*f*_f2_ [kHz]	188.43
	*Q*_a1_ [−]	148.33
	*Q*_f1_ [−]	3.95
Geometry	*L* [μm]	216
	*b* [μm]	33.8
	*h* [μm]	2.2
	*ρ*_c_ [kg/m^3^]	2298
Hydrodynamic function	*a*_1_	0.847
	*a*_2_	3.514
	*b*_1_	2.511
	*b*_2_	2.426
	Re_air_ [−]	9.07
	Re_water_ [−]	55.17
	*Γ*_r_(Re_air_) [−]	2.01
	*Γ*_r_(Re_water_) [−]	1.32
	*Γ*_*i*_(Re_air_) [−]	1.10
	*Γ*_i_(Re_water_) [−]	0.38
Spring constant	*k* [N/m]—this approach	2.165
	*k* [N/m]—from Sader	2.206

In addition to the resonance frequencies, the calculations relied on the geometrical parameters of the cantilever. These were measured by using optical microscopy images provided by Nanosurf AG. It was found that the length (*L*) of the cantilever deviated by 8% from the nominal 200 μm provided by the manufacturer (Table 1). At the end of the fabrication process, the micropipette cantilevers were attached to a plastic chip holder, and the precision of this process determined the actual length of the cantilever (e.g., the distance between the tip and the base, where the cantilever is glued to the plastic base). For the investigated cantilevers, we found that this position offset could reach ~20 μm and that the length of the cantilevers was usually longer than the nominal 200 μm. Considering that the resolution and precision of the microtechnology used for the fabrication of the cantilevers were at least an order of magnitude better, we could conclude that this positioning error could be the factor that primarily determined the resonance frequencies and thus the spring constant of the cantilevers.

For the cantilever height (*h*), a nominal value of 2.2 μm, provided by the manufacturer, was used. For width, an effective value of 33.8 μm was considered, which accounts for the wings at the two sides of the cantilever (as shown in Fig. 1d). The effective density (${\rho }_{{\rm {c}}}$) was calculated from a multiphysics model constructed with the same geometrical parameters and a microfluidic channel filled with water. The channel geometry was provided by the manufacturer: channel height of 1 μm, channel width of 30 μm, wall thickness of 600 nm, pillar diameter of 3 μm and pillar spacing of 11 μm.

By using the calibration strategy discussed in the section “Experimental”, the resulting spring constant was determined to be 2.165 N/m, which was only 1.85% smaller than the 2.206 N/m that was obtained by using the built-in calibration of the Nanosurf instrument, which relied on the Sader method. Although for the first look, this can be a positive sign, it can also show that the good agreement was mostly circumstantial.

There are four main differences between the two methods that directly affected the determined spring constants with the Sader method (Eq. (1)): (1) the quality factor (*Q*) and (2) the normalized effective mass of the cantilever (*M*_e_), (3) the geometrical parameters (*b* and *L*) and (4) the hydrodynamic function. Regarding the quality factor, due to the low noise level of the Nanosurf DriveAFM instrument, we can consider its effect negligible. As we mentioned before, although the effective mass of the micropipette cantilevers was not previously determined experimentally, the value established for long rectangular cantilevers (0.243) was used by the built-in software. We could determine *M*_e_ by using the spring constant obtained by our proposed method (by Eq. (6)) and the actual mass of the cantilever (*m*), as in Eq. (11). By using the measured parameters in Table 1 (with the exception of ${\rho }_{{\rm {c}}}$, which was determined by constructing the physical model of the cantilever and integrating its volume), *M*_e_ resulted in 0.25, which is only a 2.9% difference.

11 $${M}_{{\rm {e}}}=\frac{k}{m{\omega }_{{\rm {a}}}^{2}}=\frac{k}{{Lbh}{\rho }_{{\rm {c}}}{\omega }_{a1}^{2}}$$

The differences in the nominal and actual geometries involved both *L* and *b*. As mentioned before, based on optical microscopy investigations, the cantilever’s length was longer than the nominal 200 μm, while the width was smaller than 36 μm, considering the wings. The differences in the hydrodynamic function could be explained by looking at Fig. 4, where the real and imaginary parts of $\varGamma$ are plotted as a function of the Reynolds number for the measured cantilever. The dots represent the directly calculated values based on the first four modes of the measured resonance frequencies in air and water by using Eqs. (4), (5), and (10), and the curves represent different regressions of the hydrodynamic function.

The real part of the hydrodynamic function (${\varGamma }_{{\rm {r}}}$) of the measured FluidFM micropipette cantilever deviated from the ideal, infinitely long bulk cantilever, represented by the red curve, which used the regression from Eq. (2) with the regression coefficient provided by Maali (*a*_1_ = 1.0553; *a*_2_ = 3.7997; *b*_1_ = 3.8018; *b*_2_ = 2.7364)^37^). Using the same regression (Eq. (2)) but determining the coefficients as described in the section “Experimental” with Eqs. (8) and (9) resulted in the black curve; the coefficients are given in Table 1. Interestingly, the differences in ${\varGamma }_{i}\left(\mathrm{Re}\right)$ were minimal in the *Re* range of water (Re = 55) for the two regressions (the Maali regression provides a +7.7% higher ${\varGamma }_{i}$). Since the Sader method used this ${\varGamma }_{i}\left({\mathrm{Re}}_{{{\rm {water}}}}\right)$ for the determination of the spring constant (see Eq. (1)), the significant differences in the real part of the hydrodynamic function did not affect the calculations.

Based on this, we can conclude that the errors of the different multiplication factors in Eq. (1) compensated for each other when calculating the spring constant with the Sader method (*M*_e_: −2.9%, *L*: −8%, *b*: +6.5%, *Γ*_*i*_: +7.7), which cumulatively accounted for the relatively small difference in the obtained spring constants by using the two methods (see Table 1).

Notably, for the higher resonance modes (namely, 3 and 4), the measured ${\varGamma }_{{\rm {r}}}\left(\mathrm{Re}\right)$ values began to deviate from the regressions based on Eq. (2). This deviation could be taken into account by modifying Eq. (2) by adding another component, as in Eq. (12). As shown in Fig. 4a), this resulted in a nearly perfect fit (*R*^2^ = 0.998).

12 $${\varGamma }_{{\rm {r}}}\left(\omega \right)={a}_{1}+{a}_{2}\left(\frac{\delta }{b}\right){=a}_{1}+\frac{{a}_{2}}{\sqrt{\mathrm{Re}}}+{a}_{3}\mathrm{Re}$$

Based on Eq. (12), both Eqs. (6) and (7) could be reformulated to incorporate the new component in the regression, represented by ${a}_{3}$. However, without going into the details, the resulting spring constant differed by only 0.07% from that obtained by the method using Eq. (2) with the regression coefficients from Eqs. (8) and (9). The results from Fig. 4a confirmed this, as the regressions provided by Eqs. (2) and (12) did not significantly differ in the range of water where our calculations were performed.

Conclusively, the hydrodynamic behavior of the FluidFM micropipette cantilevers differed from the ideal, infinitely long bulk cantilever; however, in our range of possible applications, the regressions obtained by Eqs. (2) and (3) provided an adequate approximation. However, for calibration purposes, the regression coefficients of the real part (*a*_1_, *a*_2_) were calculated from Eqs. (8) and (9).

### Generalized hydrodynamic function and a simplified calibration method

For instruments that have the possibility (bandwidth) to measure the first two resonance modes in air and water, the calibration strategy discussed in the section “Determination of the hydrodynamic function, the complete calibration method and its comparison with the Sader method” could be used. However, the limited bandwidth of the robotic FluidFM did not permit the determination of the hydrodynamic function for individual cantilevers. For these systems, we proposed a simplified calibration approach that relied only on the first measured resonance modes in air and water and the generalized hydrodynamic function determined based on the nominal geometrical parameters of the cantilevers.

To obtain this generalized hydrodynamic function and the regression coefficients of its real part, three cantilevers were independently measured, and ${a}_{1}$ and ${a}_{2}$ were determined as discussed in the section “Determination of the hydrodynamic function, the complete calibration method and its comparison with the Sader method”.

**Fig. 5:**
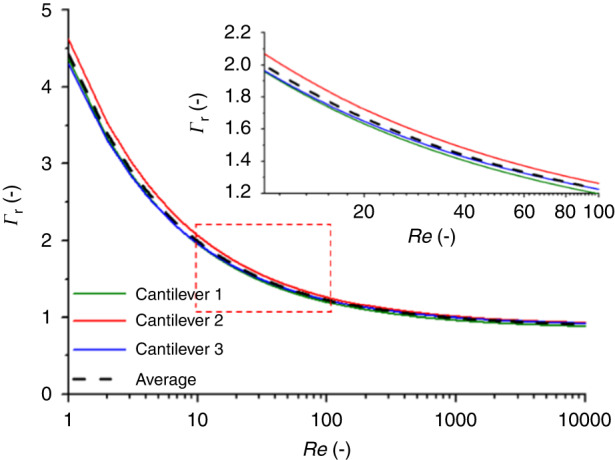
Variation of the hydrodinamic function among tested cantilevers. Real part of the hydrodynamic function for the three tested cantilevers (Table 2) and their average

As shown in Table 2 and in Fig. 5, the real parts of the hydrodynamic functions of these cantilevers were quite close to each other, and the spread of the curves was the effect of the geometrical variations of the cantilevers (expressed through the variations in the resonance positions). We could define the generalized hydrodynamic function as the average of these curves, with regression coefficients of *a*_1_ = 0.874 and *a*_2_ = 3.551. For calibration with our proposed method, only the real part of $\varGamma$ was of interest.

**Table 2:** The first two resonance peaks (in air and water), length, and obtained spring constants with the three different methods discussed in the text for the three tested cantilevers

	Cantilever 1	Cantilever 2	Cantilever 3^a^
*f*_a1_ [kHz]	77.07	81.41	78.98
*f*_a2_ [kHz]	475.91	506.25	485.00
*f*_f1_ [kHz]	27.40	28.35	27.89
*f*_f2_ [kHz]	188.43	196.42	189.50
*L* [μm]	216	210	215
*a*_1_	0.847	0.890	0.885
*a*_2_	3.514	3.727	3.411
*k* [N/m]—*Method 1*	2.165	2.360	2.263
*k* [N/m]—*Method 2*	2.219	2.294	2.292
Rel. error [%]	2.50	−2.79	1.30
*k* [N/m]—*Method 3*	2.055	2.174	2.132
Rel. error [%]	−5.09	−7.86	−5.77

^a^Cantilever 3 was measured by using a JPK NanoWizard instrument

Altogether, there are three possibilities to utilize the calibration approach elaborated in the section “Experimental”:

*Method 1* relies on all four resonance frequencies (the first two modes in air and in water) and on the actual geometrical parameters. This approach can be used where the instrument permits the measurement of all four required resonance positions, and the actual geometry (most importantly the length) of the cantilever can be estimated based on optical microscopy images. This complete method is used to initially determine the actual hydrodynamic function and its regression coefficients by solving Eqs. (8) and (9) and then use the regression coefficients to determine the spring constant from Eq. (6).

*Method 2* uses the regression coefficients of the generalized hydrodynamic function (*a*_1_ = 0.874; *a*_2_ = 3.551), the actual cantilever geometry, and only the first resonance positions measured in air and water to solve Eq. (6). This method is suitable for instruments where the bandwidth limits the determination of higher resonance modes (e.g., for the robotic FluidFM), and the cantilever length can be estimated from optical images.

If for some reason the actual cantilever geometry cannot be measured, *Method 3* can be used to solve Eq. (6) with the generalized regression coefficients (*a*_1_ = 0.874; *a*_2_ = 3.551) and nominal geometrical parameters (*L* = 200 μm, *b* = 33.8 μm, *h* = 2.2 μm).

Table 2 compares the performance of the two simplified approaches with the complete method for the three tested cantilevers. *Method 2* yielded spring constants with a relative error below ±3% compared to *Method 1*. This relative error increased to ±8% with *Method 3* by omitting the actual geometrical parameters. Notably, the Sader method also relied on these geometrical parameters (see Eq. (1)), and the built-in calibration software of the robotic FluidFM did not consider their actual values, and the nominal parameters were also used.

### Dependence on the laser spot position

In our previous work, we demonstrated that with the robotic FluidFM instrument, the determination of the spring constant was strongly dependent on the position of the laser spot on the back of the cantilever^14^. This was shown to be the effect of noise and the variation in the determination of the quality factor at the different positions.

**Fig. 6:**
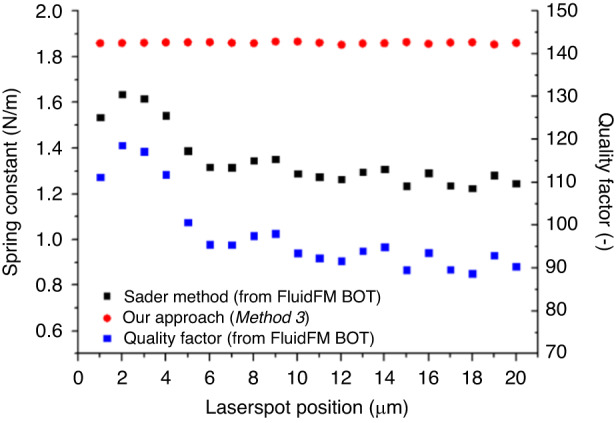
Spring constant of a micropipette cantilever as a function of the laser spot’s position along the backside of the cantilever (from the tip), measured with the robotic FluidFM instrument. Since the built-in Sader method relies on the quality factor, its variance along the length of the cantilever is also given

To demonstrate the applicability of the calibration strategy proposed in this study, we repeated the experiment in our previous work and determined the spring constant of a micropipette cantilever by changing the laser spot’s position in 1 μm increments along its backside, from tip to base (Fig. 6).

The spring constants were determined by the instrument’s built-in calibration software, which used the Sader method, and *Method* 3 was used with our approach. This method was selected for two reasons: first, as mentioned before, the instrument could only obtain the first modes of resonance in air and water and second, since the built-in Sader method utilized the nominal geometrical parameters, these parameters were also calculated for the sake of comparison.

The resonance frequencies were measured in air and in water in the exact same laser spot positions, while the channel was filled with water. The resonance frequencies were very stable (averages: ${f}_{{\rm {a}}}=78.245\,{\rm {{kHz}}}$; ${f}_{{\rm {f}}}=28.179\,{\rm {{kHz}}}$), and their deviation was in the 10 Hz (0.01%) range. Thus, the resulting spring constant from Eq. (6) was also very stable as a function of the laser spot position (average: $k=1.856\,{\rm {{N}/m}}$), and its deviation was ~0.2%.

Moreover, the spring constants were directly determined by the robotic FluidFM instrument and greatly varied with the position. The variance was the direct consequence of the improper *Q*-factor determination: here, the *Q*-factor varied between 89 and 119, depending on the position. As previously shown, due to the extensive noise (as shown in Fig. 1), the *Q*-factor could be both over- and underestimated by the used FluidFM instrument (see for example, Fig. 7 in ref. ^14^). As a result, *k* varied between 1.223 and 1.635. Additionally, the obtained *Q*-factors were significantly smaller than those obtained with the ultralow noise Nanosurf instrument (~150), which could account for the smaller spring constant values provided by the built-in software of the FluidFM instrument. This relative error could be as high as −34%, depending on the position. The difference between the two calibration methods was much higher than what was experienced with the lower-noise Nanosurf and Bruker instruments (see Table 1). Evidently, our approach based on Eq. (6) was more robust than the Sader method due to its indifference to the *Q*-factor and its noise.

### Further fine-tuning

Although the calculated hydrodynamic function for the micropipette-type FluidFM cantilevers and the proposed calibration methods have been demonstrated to provide reliable results with the tested FluidFM instruments, the model can be further refined. For example, the effect of reflective metallic coating was not considered in our current investigation. The omission was partially due to the lack of proper information on the coating, where the manufacturer only stated that the cantilevers were coated with a 40 nm thick Cr + Au layer; however, the exact composition was not specified. Additionally, the length of the coating layer was found to vary.

To estimate the effect of this metallic coating, we used the measured parameters of Cantilever 1 from the section “Generalized hydrodynamic function and a simplified calibration method”. Based on optical microscopy images, we assumed the coating to be 180 μm long (shorter than the nominal length of the cantilever), 33.8 μm wide (same as the nominal length of the cantilever) and 40 nm thick (as per the manufacturer’s specification) with an effective density and mass of 16,000 kg/m^3^ and 3.89 ng, respectively: this corresponded to a Cr:Au composition of 1:3. For the cantilever, we retained the original density and height parameters in Table 1 (${\rho }_{{\rm {c}}}$ = 2298 kg/m^3^ and *h* = 2.2 μm) and solved the equation system of Eq. (7) (for *n* = 1, 2). We considered the effect of the metallic layer as an added mass that scaled the measured resonance frequencies as per the well-known Eq. (13)^37^.

13 $${f}_{{{\rm {res}}}}\cong \frac{{f}_{{\rm {m}}}}{\sqrt{\frac{{m}_{{\rm {c}}}}{{m}_{{\rm {c}}}+{m}_{{{\rm {add}}}}}}}=\frac{{f}_{{\rm {m}}}}{\beta }$$

Here, ${m}_{{\rm {c}}}$ = 36.9 ng and is the mass of the cantilever filled with water, without the metallic layer; ${f}_{{{\rm {res}}}}$ is its theoretical resonance frequency; and ${f}_{{\rm {m}}}$ is the actual, measured resonance frequency of the cantilever, which can be scaled by estimating ${m}_{{\rm {{add}}}}$, the added mass from the metallic layer. In our example, the mass of the metallic layer was estimated as 3.89 ng, resulting in a frequency scaling factor ($\beta$) of 0.9511. Solving Eq. (7) by using the geometrical parameters and density of the cantilever from Table 1 with the scaled frequencies resulted in regression coefficients of *a*_1_ = 0.8469 and *a*_2_ = 3.6033 and a spring constant of 2.393. The regression coefficients were very close to the values in Table 1, calculated without considering the mass of the metallic layer. However, as expected, the spring constant increased by 10.5%. We recommend that users consider the effect of the reflective coating based on the optical microscopy images made on the individual cantilevers. Notably, our method could also be combined with other approaches aiming to fine-tune the spring constant determination of AFM and FluidFM cantilevers, as those listed in ref. ^45^.

## Conclusions

A method was presented to experimentally determine the real and imaginary parts of the hydrodynamic function of FluidFM micropipette-type cantilevers. The hydrodynamic behavior of the FluidFM micropipette cantilevers significantly differed from the ideal, infinitely long bulk cantilever. Using the regression coefficients of the hydrodynamic function’s real part, an approach was implemented for the spring constant calibration, eliminating the cantilever’s *Q*-factor and effective mass from the calculations. The complete calibration method relied on the determination of the hydrodynamic function based on the first two resonance positions measured in air and water and on the estimation of the actual geometrical parameters (length, width) of the used cantilever. A simplified method was also provided that only required the first resonance frequencies of the cantilever in air and water, which could be conveniently measured with the used robotic FluidFM device. Our proposed method yielded reliable and precise spring constant values, with variation below 0.2% for a given cantilever, which did not depend on the laser spot’s position on the back side of the cantilever. The implementation of our proposed method and formulas could significantly increase the reliability of force measurements with FluidFM.
